# Tumor innervation and clinical outcome in pancreatic cancer

**DOI:** 10.1038/s41598-021-86831-w

**Published:** 2021-04-01

**Authors:** Aysha Ferdoushi, Nathan Griffin, Mark Marsland, Xiaoyue Xu, Sam Faulkner, Fangfang Gao, Hui Liu, Simon J. King, James W. Denham, Dirk F. van Helden, Phillip Jobling, Chen Chen Jiang, Hubert Hondermarck

**Affiliations:** 1 School of Biomedical Sciences and Pharmacy, Faculty of Health and Medicine, University of Newcastle, Callaghan, NSW 2308 Australia; 2 Hunter Medical Research Institute, University of Newcastle, New Lambton, NSW 2305 Australia; 3 Department of Biotechnology and Genetic Engineering, Mawlana Bhashani Science and Technology University, Tangail, 1902 Bangladesh; 4 School of Population Health, Faculty of Medicine, University of New South Wales, Sydney, NSW 2052 Australia; 5 Department of Biochemistry and Molecular Biology, School of Laboratory Medicine, Bengbu Medical College, Bengbu, 233030 People’s Republic of China; 6 School of Medicine and Public Health, University of Newcastle, Callaghan, NSW 2308 Australia

**Keywords:** Cancer, Oncology

## Abstract

Pancreatic cancer is a highly aggressive malignancy characterized by poor survival, recurrence after surgery and resistance to therapy. Nerves infiltrate the microenvironment of pancreatic cancers and contribute to tumor progression, however the clinicopathological significance of tumor innervation is unclear. In this study, the presence of nerves and their cross-sectional size were quantified by immunohistochemistry for the neuronal markers S-100, PGP9.5 and GAP-43 in a series of 99 pancreatic cancer cases versus 71 normal adjacent pancreatic tissues. A trend was observed between the presence of nerves in the tumor microenvironment of pancreatic cancer and worse overall patient survival (HR = 1.8, 95% CI 0.77–4.28, *p* = 0.08). The size of nerves, as measured by cross-sectional area, were significantly higher in pancreatic cancer than in the normal adjacent tissue (*p* = 0.002) and larger nerves were directly associated with worse patient survival (HR = 0.41, 95% CI 0.19–0.87, *p* = 0.04). In conclusion, this study suggests that the presence and size of nerves within the pancreatic cancer microenvironment are associated with tumor aggressiveness.

## Introduction

The involvement of nerves in cancer progression is increasingly reported^1^. In animal models of prostate^2^ and gastric^3,4^ cancer, it has been shown that the infiltration of new nerves in the tumor microenvironment is necessary to cancer growth and metastasis and that denervation strongly inhibits tumor progression. Denervation has also been reported to reduce tumor incidence and tumor size in animal model of prostate cancer^5^. In skin cancer, sensory nerve denervation also blocks tumor initiation^6^. The impact of nerves appears to be due to the release of neurotransmitters by nerve endings in the tumor microenvironment, that stimulate neurotransmitter receptors in cancer and stromal cells, resulting in cancer growth and dissemination^7^. For instance, muscarinic receptors on gastric cancer stem cells activate stem cell expansion^3,4^ and in prostate cancer norepinephrine released by adrenergic nerves stimulate an angiogenic switch^8^. In addition, exosomes released by prostate cancer cells have been reported to induce and potentiate tumor innervation^9^. In colorectal cancer, neurogenesis has been demonstrated as an indicator of poor survival, recurrence and independent prognostic factor for poor outcomes^10,11^. Nerves of the pancreatic intraepithelial neoplasm (PanIN) microenvironment have been shown to promote oncogenesis, via direct signaling to neoplastic neuroendocrine cells capable of trophic influences^12^.

In pancreatic cancer (PC), sensory^13^ and adrenergic^14^ nerves activate tumor progression, whereas cholinergic nerves are inhibitory^15^. However, most data has been acquired from animal models and the impact of innervation in human PC is unclear. The invasion of nerves by cancer cells, termed perineural invasion (PNI), has been extensively described in PC and is generally associated with increased tumor aggressiveness^16^. Despite this, it is unclear if the increased PNI in PC is primarily due to increased invasiveness of PC cells or to the increased infiltration of new nerves in the tumor microenvironment.

In the present study, we have analyzed nerve infiltration in the tumor microenvironment of a cohort of human PC. The data reveal an increased innervation as well as an increase in nerve size, which are both associated with poor clinical outcomes. These findings point to tumor innervation as a risk factor in PC and suggest that increased nerve infiltration is a primary event leading to higher PNI observed in PC.

## Materials and methods

### Pancreatic tissue samples

High-density tissue microarrays (TMA) were obtained from US Biomax Inc. (Maryland, USA). The TMA used (catalogue number: HPan-Ade170Sur-01) included a total of 99 PCs and 71 adjacent normal pancreatic tissues (NATs). Pancreatic ductal adenocarcinoma (PDAC) was the major subtypes (99%), minor subtypes included adenosquamous carcinoma (6%), ductal adenocarcinoma/partly mucinous adenocarcinoma (4%), ductal adenocarcinoma/adenosquamous carcinoma (1%) (Supplementary Table S1). The available clinicopathological information was as follows: patient sex and age, histological subtypes, tumor grade, stage, size, lymphatic metastasis status, survival status, surgery date, visit date and survival month. No information on treatment was available. Supplementary Table S1 summarizes the clinicopathological characteristics and distribution of events in the 99 patient population. US Biomax Inc. quality controls are described as follows. Each single tissue spot on every array slide was individually examined by pathologists certified according to WHO published standardizations of diagnosis, classification and pathological grade. Each specimen collected from any clinic was informed consented to by both hospital and individual. The study was approved by the Human Research Ethic Committee of the University of Newcastle (HREC reference: H-2012-0063) and all experiments were performed in accordance with relevant guidelines and regulations.

### Immunohistochemistry

To investigate the presence of nerves, immunohistochemistry (IHC) against the neuronal markers S-100, the pan-neural marker protein gene product 9.5 (PGP9.5) and the growth-associated protein 43(GAP-43) was performed as described previously^17^. Following deparaffinization and rehydration of the TMA slides using standard procedures, heat induced epitope retrieval was carried out in a low pH, citrate based antigen unmasking solution (catalogue number H-3300, Vector Laboratories, California, USA) by a decloaking chamber (Biocare, West Midlands, UK) at 95 °C for 30 min and 90 °C for 10 s. IHC was then performed using an ImmPRESS HRP IgG (peroxidase) Polymer Detection Kit (catalogue number MP-7405, Vector Laboratories), as per the manufacturer’s recommendations. Briefly, after inactivation of endogenous peroxidases with 0.3% H_2_O_2_, and blocking with 2.5% horse serum, primary antibodies (anti-S-100, 1:500 dilution, catalogue number Z0311, Dako, Australia; anti- PGP9.5, 1:200 dilution, catalogue number ab15503, Abcam, Australia; anti-GAP-43, 1:250 dilution, catalogue number ab75810, Abcam, Australia) followed by secondary antibodies were applied to the sections and revealed with DAB Peroxidase (HRP) Substrate Kit (catalogue number SK-4100, Vector Laboratories). Finally, TMA slides were counterstained with hematoxylin (Gill’s formulation, Vector Laboratories), dehydrated and cleared in xylene before mounting in Ultramount #4 mounting media (Thermo Fisher Scientific, Victoria, Australia).

### Quantitative analysis of neural tissue

Following IHC staining, slides were digitized using the Aperio AT2 scanner (Leica Biosystems, Victoria, Australia)^17^. Identification and quantification of nerves were performed manually and verified by an expert pathologist. The number of nerves that were positive for the nerve markers were also counted for each case. A clinical sample was considered nerve positive when nerves could be detected by all three markers (S-100, PGP9.5, GAP-43). Nerve size (cross-sectional area) was measured using the Aperio’s ImageScope software (v12.4.0.5043, Leica Biosystems).

### Statistical analysis

Prism statistical analysis software (version 8.0, GraphPad Software Inc., La Jolla, California, USA) and STATA/SE 14 (StataCorp, USA) were used to conduct all statistical analyses. Univariate and multivariate Cox proportional hazards regression models were performed to examine the associations between nerve infiltration status and survival. Correlation between nerves and other pathological variables were performed using a chi-squared test. To compare differences of means between two groups of replicates an unpaired, two-tailed t-test was performed unless indicated otherwise. The reported *p*-values were considered statistically significant if they were less than 0.05. Overall survival (OS) analysis was performed by the Kaplan–Meier method. OS was calculated as the time from the date of diagnosis to the date of death. Log-rank test was used to examine the statistical significance on survival distributions by nerve infiltration. Hazard ratio (HR) computed by the Mantel–Haenszel method was used to measure the rapidity of subject death.

## Results

### Nerve detection in PC and normal pancreatic tissues

**Figure 1:**
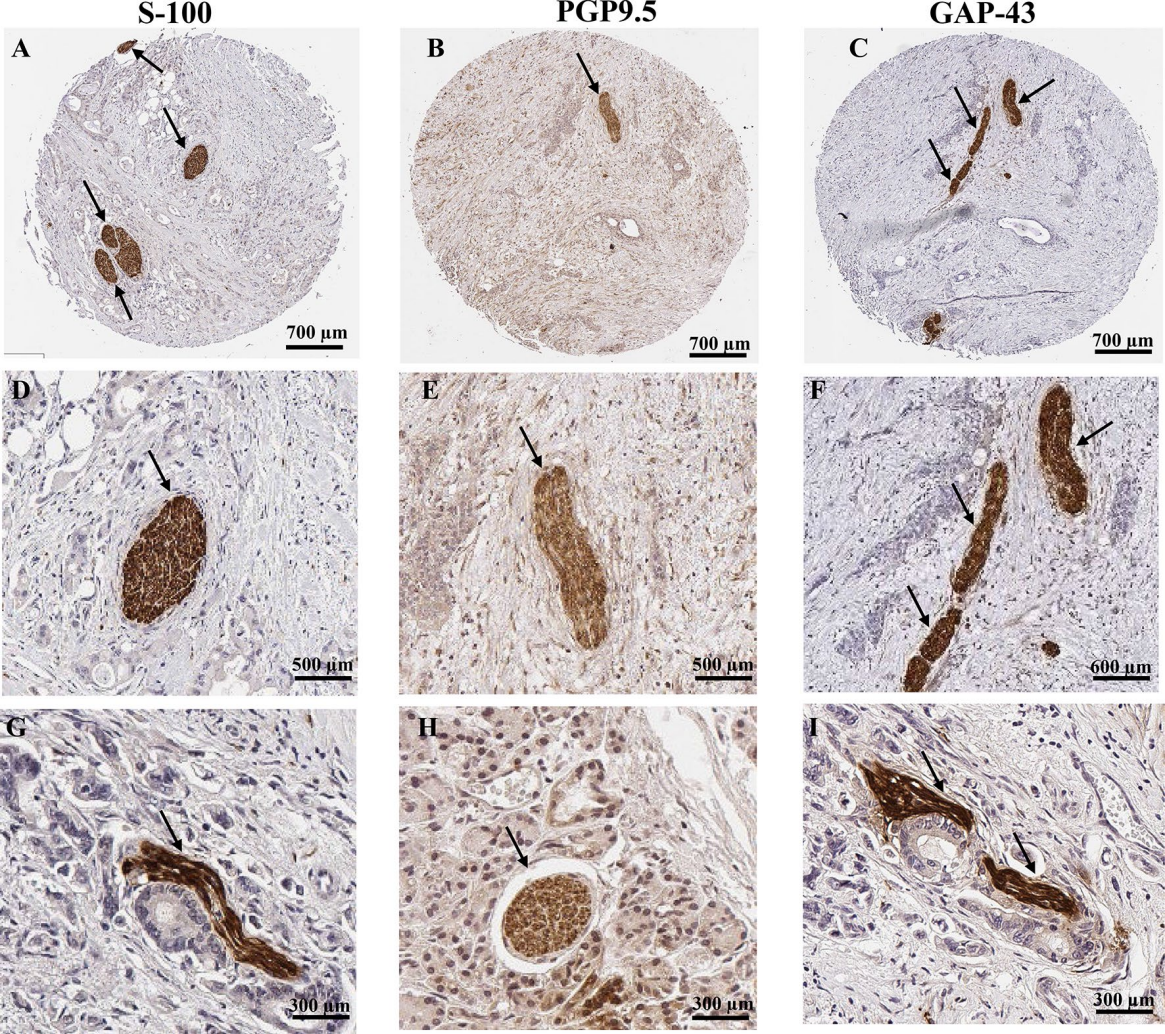
Nerves in the tumor microenvironment of PC. Peripheral nerves in human PC and normal adjacent pancreatic tissues were stained using IHC for the neuronal markers S-100, PGP9.5 and GAP-43; counterstaining was performed with hematoxylin. Representative images of nerve sections from PC tissues (**A**–**C** entire core; **D**–**F** higher magnification of nerves in **A**–**C** in the tumor microenvironment) and normal adjacent pancreatic tissues (**G**–**I**). Stained nerves are indicated by black arrows. *PC* pancreatic cancer.

Typical morphological features corresponding to nerves were observed by IHC staining for the neuronal markers S-100, PGP9.5 and GAP-43 (Fig. 1). A total of 10% of PC and 13% of adjacent normal pancreatic tissue were found to be infiltrated by nerves (Table 1). Nerves were typically found in the periphery of the tumors (Fig. 1A) or invading deep inside the tumor (Fig. 1B,C). Hematoxylin and Eosin (H&E) stained images for the same cores are shown in Supplementary Fig. S2.

**Table 1:** Nerve infiltration in the pancreatic tumor microenvironment and its correlation with clinicopathological variables.

Parameters	Nerve negative	Nerve positive	Undefined	*p*-value
**Pathology description**	**N (%)**			
Normal (n = 71)	20 (28)	3 (13)	48 (68)	0.74
Cancer (n = 99)	47 (47)	10 (10)	42 (42)	
**Cancer histological subtype**				
Ductal adenocarcinoma (n = 88)	40 (45)	10 (11)	38 (43)	0.33
Others (n = 11)	7 (64)	0 (0)	4 (36)	
**Sex**				
Male (n = 63)	26 (41)	5 (8)	32 (51)	1.0
Female (n = 36)	21 (58)	5 (14)	10 (28)	
**Age (years)**				
≤ 50 (n = 16)	8 (50)	3 (19)	5 (31)	0.38
> 50 (n = 83)	39 (47)	7 (8)	37 (45)	
**Tumor grade**				
G1 (n = 11)	4 (36)	3 (27)	4 (36)	0.09
G2 + G3 (n = 88)	43 (49)	7 (8)	38 (43)	
**Tumor stage**				
0 + I (n = 40)	19 (47)	4 (10)	17 (42)	1.0
II + IV (n = 59)	28 (47)	6 (10)	25 (42)	
**Tumor size**				
T1 + T2 (n = 78)	37 (47)	8 (10)	33 (42)	1.0
T3 (n = 20)	10 (50)	2 (10)	8 (40)	
**Lymphatic metastasis status**				
Negative (n = 50)	25 (50)	4 (8)	21 (42)	0.45
Positive (n = 43)	18 (42)	5 (12)	20 (46)	
**Survival status (months)**				
Survival > 10 months (n = 49)	26 (53)	3 (6)	20 (41)	0.17
Survival ≤ 10 months (n = 50)	21 (42)	7 (14)	22 (44)	

*p*-values were calculated by the chi-squared test between nerve positive *versus* nerve negative samples. A tumor was considered positive only when nerves could be confirmed by using 3 neuronal markers (S-100, PGP9.5 and GAP-43).

### Nerve infiltration is associated with worse prognosis in PC

**Figure 2:**
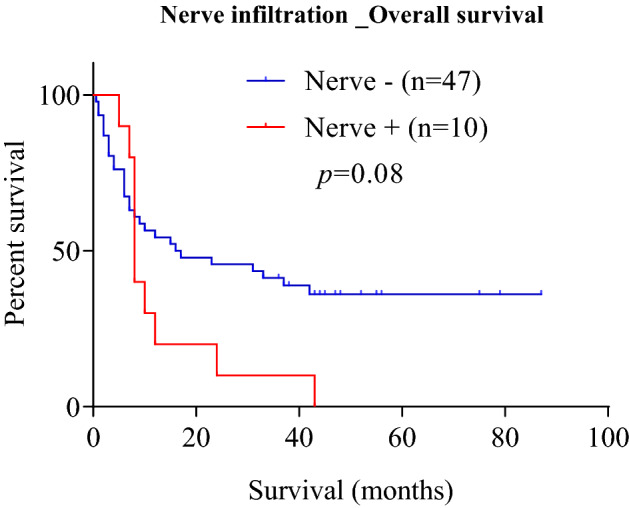
The presence of nerves in the tumor microenvironment of PC is associated with poor survival. Kaplan–Meier survival curve comparing cases positive versus negative for nerve infiltration. Patients with nerve infiltration harbor the worst prognosis i.e. had shorter overall survival compared to without nerve infiltration. The results of comparative analyses are presented in Table 2. *PC* pancreatic cancer.

To investigate the potential association between nerve infiltration and clinicopathological outcomes, each clinical case was classified as nerve positive *versus* nerve negative. A complete summary of clinicopathological associations with nerve infiltration is presented in Table 1. No statistically significant associations were found between nerve infiltration and age, sex, tumor grade, tumor stage, tumor size, tumor histological type, lymph node metastasis or patient survival (*p* > 0.05) (Table 1). A trend was observed between the overall presence of nerves in the pancreatic tumor microenvironment and worsening of patient survival (8 versus 16.5 months of survival in nerve negative and nerve positive tumors respectively), despite not reaching statistical significance (HR = 1.8, 95% CI 0.77–4.28, *p* = 0.08) (Fig. 2). The results of the univariate Cox proportional hazards regression models of examining the association between nerve infiltration and survival are shown in Table 2. Nerve positive patients had a 1.8-fold elevated risk of death compared to patients without nerve infiltration (Table 2).

**Table 2:** Univariate Cox proportional hazards regression models of the association between nerve infiltration and survival.

	Univariate model	
	HR (95% CI)	*p-*value
**Nerve infiltration**		
Negative (n = 47)	1 (reference)	0.08
Positive (n = 10)	1.8 (0.77–4.28)	

HR was calculated using univariate Mantel–Haenszel hazard model.

*HR* hazard ratio, *CI* confidence interval.

*P*-values were calculated by the log-rank test.

**Figure 3:**
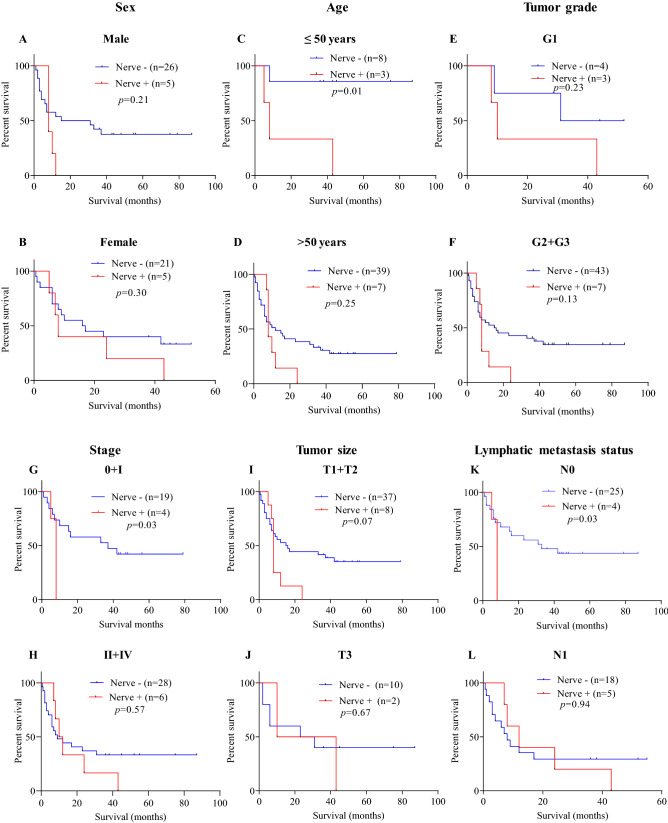
Association between the presence of nerves and overall survival in stratified patient groups. Kaplan–Meier survival curve comparing cases positive *versus* negative for nerve infiltration after patient stratification in function of sex (**A**,**B**), age (**C**,**D**), tumor grade (**E**,**F**), stage (**G**,**H**), tumor size (**I**,**J**) and lymphatic metastasis status (**K**,**L**). The results of multiple comparative analyses are presented in the Table 3.

Patients were further stratified (Fig. 3A–L) based on clinicopathological parameters and association with nerve infiltration was investigated using Kaplan–Meier survival analysis (Table 3). In terms of age, for patients aged less than 50 years, nerve infiltration was associated with significantly poorer prognosis (*p* = 0.01, Fig. 3C) compared to those without nerve infiltration, equating to a ninefold elevated risk of death (Table 3). No significant association was found between nerve infiltration and survival of patients aged more than 50 years (*p* = 0.25, Fig. 3D). In terms of stage, patients with lower stage (0 + I) and positive for nerves had poorer clinical outcomes than those negative for nerves with 3.0-fold elevated risk of death (*p* = 0.03, Fig. 3G) (Table 3). A trend was observed between nerve infiltration and shorter OS in patients with lower tumor stage (T1 + T2), but differences did not reach statistical significance (*p* = 0.07, Fig. 3I). For patients with higher (T3) tumor size, nerve infiltration had no significant association with OS (*p* = 0.67, Fig. 3J) (Table 3). In patients without lymph node metastasis (N0), the nerve infiltrated group showed worse survival compared to those without nerve infiltration (8 versus 33 months*, p* = 0.03**,** Fig. 3K) with 3.0-fold elevated risk of death (Table 3). Patients with nodal metastasis (N1) and positive for nerves had better clinical outcomes than those negative for nerves though differences did not reach statistical significance (12 versus 8 months, *p* = 0.94, Fig. 3L) (Table 3).

**Table 3:** Association between nerve infiltration and clinicopathological characteristics.

Parameters	N (%)	Median survival month	HR (95% CI)	*p*-value	N (%)	Median survival month	HR (95% CI)	*p*-value
**Sex**	**Male**				**Female**			
Nerve +ve	5 (16)	8	1 (reference)	0.21	5 (19)	8	1 (reference)	0.30
Nerve −ve	26 (84)	23	1.80 (0.54–6.04)		21 (81)	16.5	1.70 (0.51–5.59)	
**Age (year)**	** ≤ 50 year**				** > 50 year**			
Nerve +ve	3 (27)	8	1 (reference)	**0.01**	7 (15)	8	1 (reference)	0.25
Nerve −ve	8 (73)	Undefined*	9.70 (0.96–97)		39 (85)	12	1.57 (0.59–4.11)	
**Grade**	**G1**				**G2 + G3**			
Nerve +ve	3 (43)	10	1 (reference)	0.23	7 (14)	8	1 (reference)	0.13
Nerve −ve	4 (57)	41.5	2.79 (0.44–17)		43 (86)	15.5	1.81 (0.65–5.01)	
**Stage**	**0 + I**				**II + IV**			
Nerve +ve	4 (18)	8	1 (reference)	**0.03**	6 (18)	11	1 (reference)	0.57
Nerve −ve	19 (82)	37	3.04 (0.59–15)		28 (32)	9	1.29 (0.47–3.47)	
**Tumor size**	**T1 + T2**				**T3**			
Nerve +ve	8 (18)	8	1 (reference)	0.07	2 (17)	26.5	1 (reference)	0.67
Nerve –ve	37 (82)	15.5	1.95 (0.73–5.22)		10 (83)	27	1.39 (0.24–8.07)	
**Lymph node status**	**N0**				**N1**			
Nerve +ve	4 (14)	8	1 (reference)	**0.03**	5 (22)	12	1 (reference)	0.94
Nerve –ve	25 (86)	33	3.02 (0.58–15)		18 (78)	8	1.03 (0.36–2.96)	

Patients with different clinicopathological parameters were stratified as positive or negative for the presence of nerve infiltration to assess the association of nerve infiltration with clinicopathological parameters in PC.

*HR* hazard ratio, *CI* confidence interval.

*p*-values were calculated by the log-rank test. Values in bold indicate significance (*p* < 0.05). Star sign (*) denotes if more than 50% of the subjects are alive at the end of the study, then the median survival time is simply not defined/undefined. HR was calculated using univariate Mantel–Haenszel hazard model.

Together, these results suggest that nerve infiltration is a marker of worse survival among patients of lower tumor stages and sizes. In addition, nerve infiltration is a marker of poor survival among patients without lymphatic metastasis and indicates that there is a subpopulation of N0 patients which are at an increased risk of death if the tumor is infiltrated by nerves.

### Enlarged nerve cross sectional area is a feature of malignant PC and unfavorable prognosis

**Figure 4:**
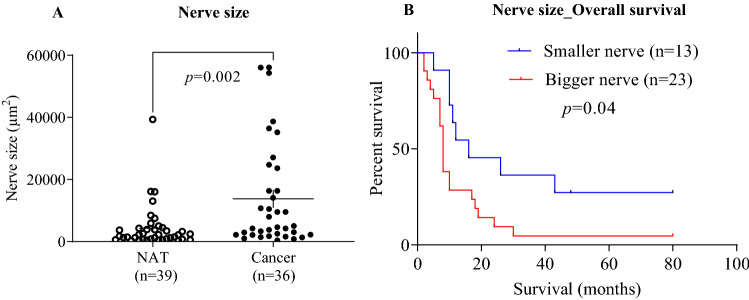
Increased nerve size is associated with pancreatic malignancy and patient survival. Pancreatic tissue sections from PC and normal adjacent tissue (NAT) were analyzed for changes in nerve size (measured by cross-sectional area). The nerve size was bigger in PC tissue than NAT (**A**). The median value of nerve area was taken as cut-off to define two groups (bigger nerve, nerve area bigger than median value and smaller nerve, nerve area smaller than median value) and used for the Kaplan–Meier analysis and the log-rank test. Bigger nerves in the tumor microenvironment was associated with worse patient survival (**B**). Survival analyses displayed a significantly shorter survival for patients with bigger nerve size than that of smaller nerve size. The results of comparative analyses are presented in Table 4A,B. *PC* pancreatic cancer.

To investigate the association between nerve size and pancreatic malignancy, the areas of intrapancreatic tumor nerves were measured by cross-sectional area (Supplementary Fig. S3) from S-100 labelled nerves. The mean nerve area was significantly higher in PC tissues compared with normal adjacent pancreatic tissue (*p* = 0.002) (Table 4A, Fig. 4A, Supplementary Fig. S4). The mean area of nerves in PC tissues was 13,756 μm^2^, which was almost four times greater than that of the normal adjacent tissue (*p* = 0.002) (Table 4A). Sex, tumor size, grade, stage or lymphatic metastasis status were not associated with nerve size in pancreatic tumors (Supplementary Fig. S5).

**Table 4:** (A) Comparison of nerve related parameters in PC tissue and normal adjacent tissue.

Variables	Number of nerves positive samples	MNA (μm^2^)	MNA (μm^2^) ± SEM	*p*-value
**(A)**				
NAT	39	4253	4253 ± 1121	**0.002**
Cancerous tissue	36	13,756	13,756 ± 2778	

Nerve size (μm^2^)	N (%)	Median survival month	HR (95% CI)	*p*-value
**(B)**				
Bigger nerve (> 3200 μm^2^)	23 (64)	8	1 (reference)	**0.04**
Smaller nerve (≤ 3200 μm^2^)	13 (36)	16	0.41 (0.19–0.87)	

Unpaired t-test was performed to compare nerve related parameters in the different groups. Data are represented as mean ± SEM. (B) Correlation between increased nerve size and patient survival. *P*-values were calculated by the log-rank test. HR was calculated using univariate Mantel–Haenszel hazard model. Values in bold indicate *p* < 0.05.

*PC* pancreatic cancer, *NAT* normal adjacent tissue, *MNA* mean nerve area (μm^2^), *SEM* standard error of mean, *HR* hazard ratio, *CI* confidence interval.

To investigate the potential association between nerve size and patient survival, the nerves in PC tissue were dichotomized into smaller nerve (area, ≤ 3200 μm^2^) and larger nerve (area, > 3200 μm^2^). This cut-off was established based on the median nerve size area (3200 μm^2^). Kaplan–Meier survival analysis demonstrated that patients with bigger nerve size were significantly associated with worse prognosis, where the median survival month for patients with bigger nerve size was lower than that of smaller nerves (8 versus 16 months) (*p* = 0.04, Fig. 4B, Table 4B).

## Discussion

PC prognosis is influenced by several clinicopathological factors including tumor size^18,19^, grade^20,21^, lymph node invasion^22^ and depth of invasion^23^. In various malignancies, tumor innervation has been reported to play a pivotal role in driving tumor growth^2,4,14^. The present study demonstrates that the presence and size of nerves in the tumor microenvironment is associated with worse patient survival in PC. It is important to distinguish between de novo innervation (new nerves), PNI, and nerve hypertrophy. The data presented indicate that the number of nerves between PC and normal adjacent tissue is the same (Tables 1 and 4). It is the size of the nerves that seems to be different between PC and normal adjacent tissues. Hence, it is likely that the phenomenon being observed is nerve hypertrophy, not de novo innervation as has been described in other tumors.

In the present study, IHC staining of the neuronal markers S-100, PGP9.5 and GAP-43 was performed to detect nerves in the microenvironment of PC. The presence or absence of nerves were investigated as well as nerve size were measured and analyzed in relation to clinical outcomes. A trend was found between nerve infiltration and poor prognosis, but statistical significance (*p* < 0.05) was not obtained. Marginal significant differences in nerve infiltration regarding OS of PC has already been reported in a study conducted by Min et al.^24^. In contrast, Iwasaki has shown that low intrapancreatic nerve numbers were correlated with shorter OS of PC patients^25^. These contradictory published results are in agreement with our own data showing only a trend (*p* = 0.08) for the association between nerve infiltration and PC survival. PC is defined as a disease of elderly populations and rarely occurs before the age of 40^26,27^. Our study demonstrates that patients aged ≤ 50-years with nerve infiltration had more chance of death compared to without nerve infiltration. This finding suggest that nerve infiltration is a prognostic factor particularly for patients diagnosed with PC at early age. Lymphatic metastasis, which develops in 60–70% patients with PC^28^, has been described as one of the most important prognostic factors^29^. Patients with surgical resection often encounter disease recurrence with a high frequency of lymph node metastases^30^. We found that patients without lymphatic metastasis but infiltrated by nerves had 4 times more chance of death compared to without nerve infiltration with a statistically significant reduction in patient survival. This finding suggests that nerve infiltration is a risk factor for poor survival among patients without lymphatic metastasis. Altogether, our study revealed that nerve infiltration might be a potential prognostic indicator in PC in the case of patients diagnosed at an early age, lower tumor stage and negative lymphatic metastases.

The median nerve area (measured cross-sectionally) was higher in cases of PC tissue compared to normal adjacent tissue. Similar neural alteration was observed by Li *et. al.*, who reported that the median number of nerves and median nerve diameters were greater in PC patients with diabetes mellitus^31^. They concluded that, for patients with hyperglycemia, nerve damage and regeneration are a simultaneous process in the tumor microenvironment of PC where abnormal expression of NGF and p75 play a pivotal role^31^. Increased hypertrophy has previously been suggested in PC^32^ and chronic pancreatitis^33,34^. A study in breast cancer revealed that thickness of tumor- related nerve fibers is significantly associated with poor differentiation, lymphatic metastasis, high clinical staging, and a triple negative subtype^35^. In our study, nerve size was correlated with patient survival but not with tumor grade, size, stage or lymphatic metastasis. It has been reported that neurotrophic factors secreted from cancer cells and other stromal cells promote neuronal hypertrophy in cancer and drive cancer progression^35,36^ and an increased production of neurotrophic factors may account for the increased nerve size that we report here. Nerve growth factor (NGF) in particular has been shown to be a driver of nerve infiltration in PC^14^ and blocking NGF signaling reduces the neural invasion potential of PC cells^37^. Anti-NGF siRNA encapsulated in nanoparticles have been shown to be able to decrease pancreatic tumor growth in the mouse^38^ suggesting that the increase in nerve infiltration and size participate in PC progression.

Together, our findings outline the clinicopathological significance of nerve infiltration in the tumor microenvironment of PC. The association of infiltrating nerves with tumor aggressiveness suggests that increased infiltration of larger size nerves might be the reason for increased PNI commonly found in PC. An interesting hypothesis that will need to be explored is that innervation in pancreatic tumors may be associated with PC chemoresistance, i.e., nerve infiltration may participate in chemoresistance. Indeed, the inhibition of NGF receptors, a driver of tumor innervation, has been shown to improve the effect of gemcitabine’s treatment (gemcitabine is a chemotherapy commonly used in PC)^14^. A recent study has also shown that tumor-infiltrating nerves contribute to treatment resistance in case of ovarian cancer^39^. Further experimental investigations are warranted to confirm this hypothesis and the potential value of using nerve infiltration as a prognostic biomarker in PC.

## Supplementary Information

Supplementary Information

## Data Availability

All data generated or analyzed during this study are included in this published article (and its Supplementary Information files).

## Abbreviations

HR: Hazard ratio
IHC: Immunohistochemistry
MNA: Mean nerve area
NAT: Normal adjacent tissue
OS: Overall survival
PC: Pancreatic cancer
PDAC: Pancreatic ductal adenocarcinoma
PNI: Perineural invasion
TMA: Tumor microarrays
PNI: Perineural invasion
