# Supplementary material for: Tumor innervation and clinical outcome in pancreatic cancer
Source: Sci Rep. 2021 Apr 1;11:7390. doi: 10.1038/s41598-021-86831-w (PMC8017010; doi:10.1038/s41598-021-86831-w)
Supplement: Supplementary file 1 — Supplementary Information [file 41598_2021_86831_MOESM1_ESM.pdf]

Tumor innervation and clinical outcome in pancreatic cancer

Aysha Ferdoushi[1,2,3], Nathan Griffin[1,2], Mark Marsland[1,2], Xiaoyue Xu[4], Sam Faulkner[1,2], Fangfang Gao[1,2], Hui Liu[5], Simon J. King[2], James W Denham[2,6], Dirk F. van Helden[1,2], Phillip Jobling[1,2], Chen Chen Jiang[2,6], Hubert Hondermarck[1,2*]

[1]School of Biomedical Sciences and Pharmacy, Faculty of Health and Medicine, University of Newcastle, Callaghan NSW 2308, Australia.

[2]Hunter Medical Research Institute, University of Newcastle, New Lambton NSW 2305, Australia.

[3]Department of Biotechnology and Genetic Engineering, Mawlana Bhashani Science and Technology University, Tangail 1902, Bangladesh.

[4]School of Population Health, Faculty of Medicine, University of New South Wales, Sydney, NSW 2052, Australia.

[5]Department of Biochemistry and Molecular Biology, School of Laboratory Medicine, Bengbu Medical College, Bengbu 233030, P.R. China.

[6]School of Medicine and Public Health, University of Newcastle, Callaghan NSW 2308, Australia.

*Correspondence: Hubert Hondermarck, School of Biomedical Sciences and Pharmacy, University of Newcastle, Callaghan NSW 2308, Australia. Tel: +61 2 4921 8830. Email: hubert.hondermarck@newcastle.edu.au

Supplementary Table S1:

Patient clinicopathological characteristics. Unless indicated otherwise, data show the number of patients in each group, with percentages given in parentheses.

Parameters	Category	N (%)
Pathology description	Normal	71 (42)
	Cancer	99 (58)
Histological subtype	Pancreatic ductal adenocarcinoma (PDAC)	88 (89)
	Adenosquamous carcinoma	6 (6)
	Ductal adenocarcinoma/partly mucinous adenocarcinoma	4 (4)
	Ductal adenocarcinoma/adenosquamous carcinoma	1 (1)
**Patients characteristics**		
Sex	Male	63 (64)
	Female	36 (36)
Age (years)	≤50	16 (16)
	>50	83 (84)
**Tumor characteristics**		
Tumor grade	G1	11 (11)
	G2+G3	88 (89)
Tumor stage	0+ I	40 (46)
	II+ IV	59 (54)
Tumor size	T1+T2	78 (80)
	T3	20 (20)
Lymphatic metastasis status	N0	50 (54)
	N1	43 (46)

Supplementary Figure S2:

H&E staining of PC tissue and adjacent normal pancreatic tissue. Images (obtained from US Biomax Inc.) of PC tissues (A, B, C, D, E, F) and adjacent normal pancreatic tissues (G, H, I) from the same cores displayed in Figure 1. Arrow heads pointing nerves (encircled, A, D), cancer cells (E, F) and acinar cells (G, H). PC, pancreatic cancer; H&E, Haemotoxylin and Eosin.

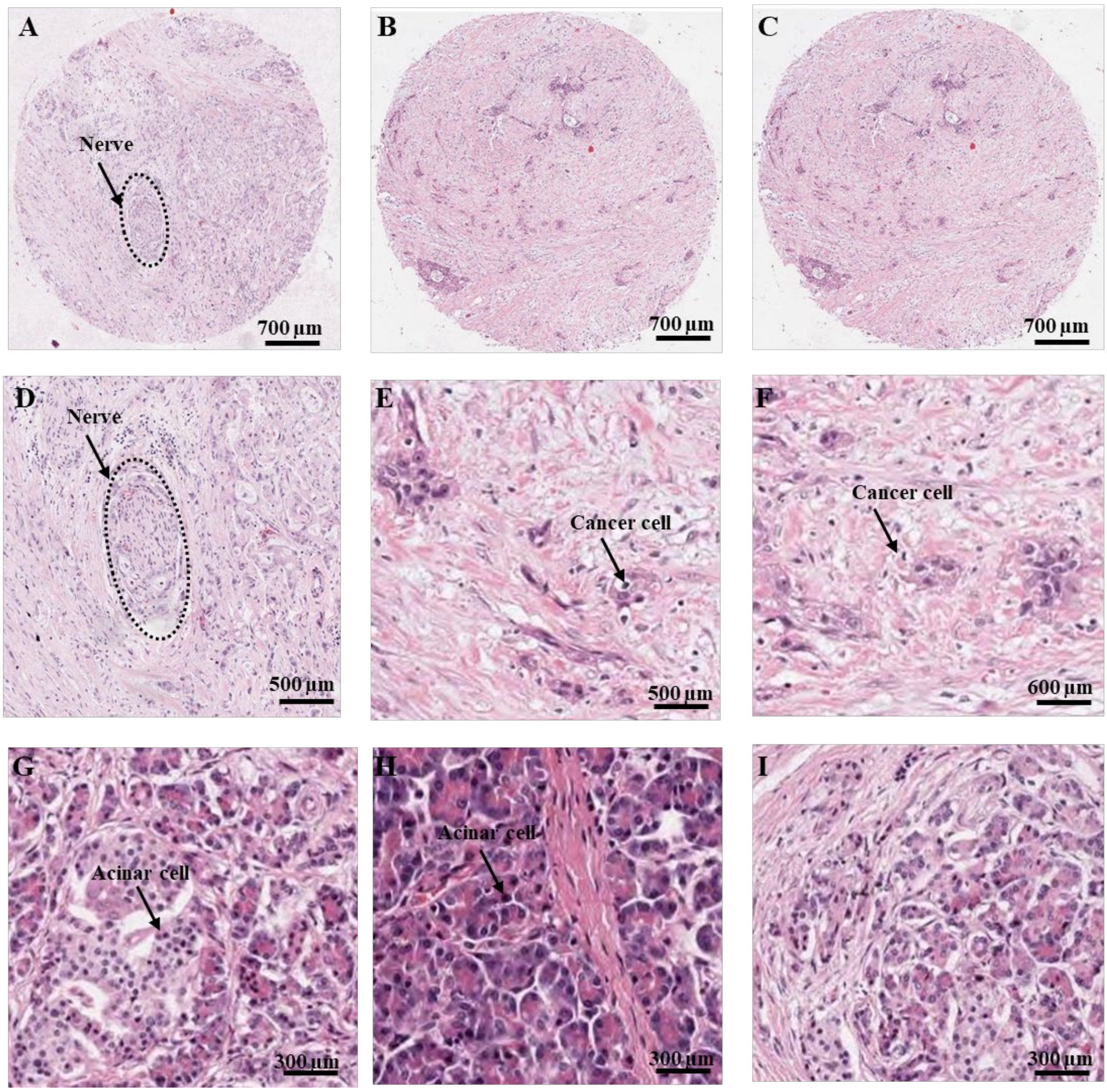

Supplementary Figure S3:

Examples of nerve measurements. Peripheral nerves were stained using immunohistochemistry for the neuronal makers S-100, PGP9.5 and GAP-43 and counter stained with haematoxylin. Nerve size was measured by cross-sectional area using Aperio ImageScope software (pen tool-F2), where each nerve section was outlined manually, and the area measurement was automatically obtained. Measurement of same image using different scale bar are shown here: (A) 500µm and (B) 100µm.

A

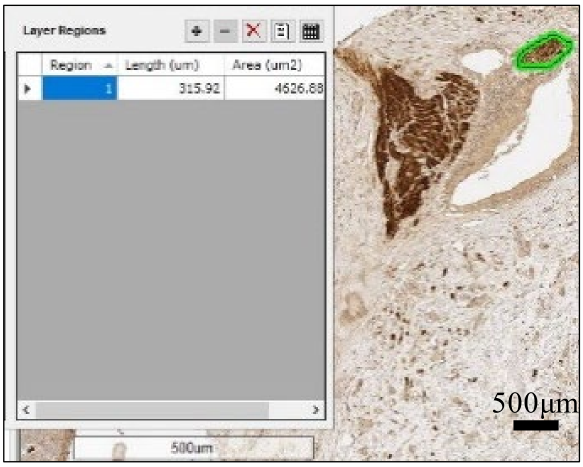

B

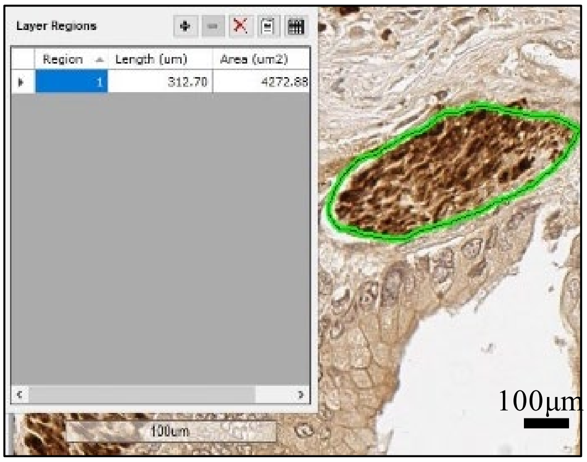

Supplementary Figure S4:

Nerve size is increased in PC tissues. Nerve tissue immunostaining in different groups. S-100 immunostaining in the PC group (A, C) and normal adjacent pancreas (B, D). Increases in the area of nerve tissues were observed in PC compared with normal adjacent pancreatic tissue. Stained nerves are indicated by red arrowheads. Scale bar = 1mm. PC, pancreatic cancer.

Pancreatic cancer tissue **Normal adjacent tissue**

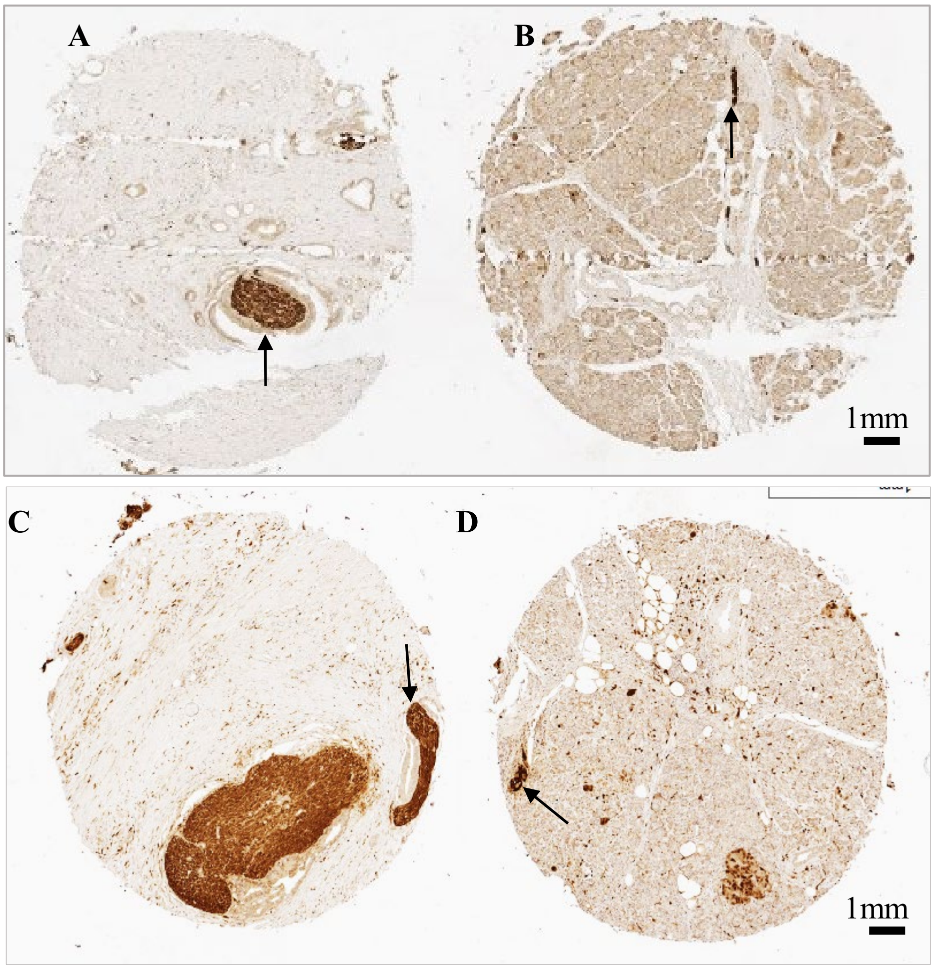

C

Supplementary Figure S5:

Correlation between nerve size and different clinicopathological characteristics. Nerve size was not associated with sex, stage, grade, tumor size or lymphatic metastasis.

Nerve size_Clinicopathological parameters

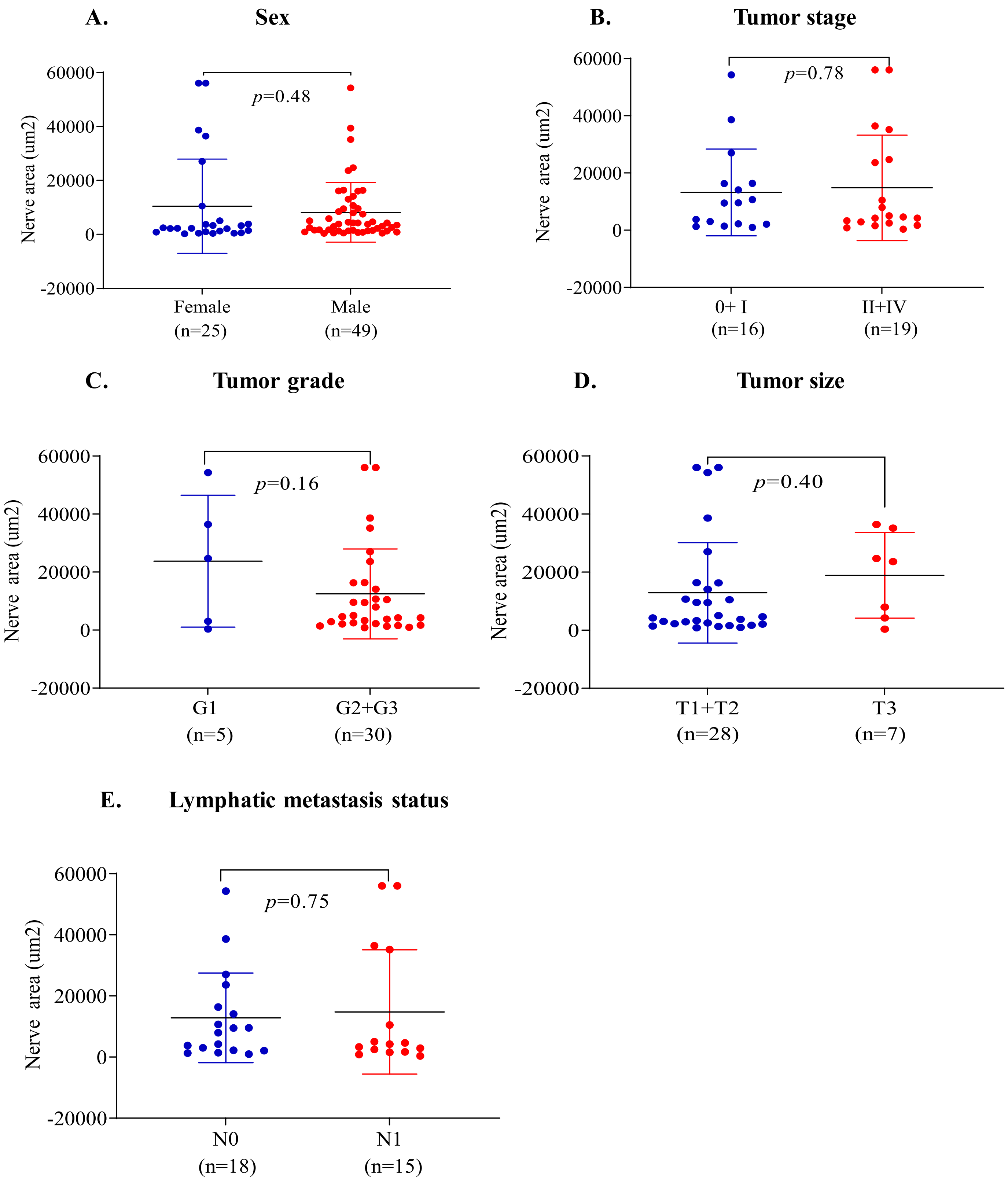

Supplementary Table S6:

Univariate and multivariate Cox proportional hazards regression models of the association between lymphatic metastasis status and survival.

	**Univariate model**		**Multivariate model**	
	**HR (95% CI)**	***p*-value**	**HR (95% CI)**	***p*-value**
**Lymphatic metastasis status**				
Negative (n=50)	1 (reference)	**0.006**	1 (reference)	0.72
Positive (n=43)	1.85 (1.19-2.88)		1.13 (0.57-2.24)	

Lymph node status is significantly associated with survival in the univariable model (HR= 1.85, p=0.006), however, no statistically significant differences were found after adjusted for age and tumor stage in the multivariable model (HR=1.13, p=0.72). HR: hazard ratio; CI, confidence interval.
